# A systemic challenge in dietetics: Methodological inadequacies, erroneous claims, and misleadinginterpretations, and transparency of post-publication scrutiny

**DOI:** 10.1177/02601060221094126

**Published:** 2022-04-13

**Authors:** Ognjen Arandjelović

**Affiliations:** 7486 University of St Andrews, North Haugh, St Andrews, KY16 9SX, UK

**Keywords:** Science communication, publication, peer review, obesity, nutrition, nutrient timing

## Abstract

**Background:** Obesity is sweeping across the developed world. Yet, the public remains largely confused when it comes to the nature of dietary habits which would serve to counteract this trend. **Aim:** I highlight the responsibility that the scientific community bears when it comes to the confusion, and explain the kind of actions that are needed if the public trust in science is to be maintained. **Methods:** Starting from an example of a recently published and prominently featured article in a leading journal, I analyse various common methodological aspects of dietetics research and the consequent claims, contextualizing this within the broader environment which includes the scientific publishing process and the mainstream media. **Results:** Methodological inadequacies, erroneous claims, and misleading interpretations of findings are often found in dietetics research, highlighting the deficiencies of the system which fails to uphold the fundamental principles of scientific inquiry. **Conclusion:** It is imperative that individual scientists speak out and challenge poor science, unsatisfactory publishing processes, and bombastic and misleading communication of research.

## Introduction

Obesity presents a major problem throughout the developed world. This is no new phenomenon – obesity has been on a sharp rise for at least a few decades (Bleich et al. 2008). The consequent health care and social costs (de Oliveira et al. 2015; Tran et al. 2013; Levy et al. 1995; Allender and Rayner 2007), and the widespread distal economic burden of the condition (Wolf and Colditz 1998, 1994), have made addressing the challenge of slowing down, stopping, or better yet reversing this trend one of the priorities of governments across the globe (Watson et al. 2018; Novak and Brownell 2012). Considering the general ethos of personal liberty, for the most part the approach has been that of ‘education’ (though scattered disincentivization measures, e.g. by means of increased taxation of certain kinds of foods, are noteworthy (Stafford 2012; Burki 2016)), that is, of attempting to increase the general awareness of the condition and to effect a change in dietary and lifestyle habits. That this effort has thus far, at best, had highly limited effect is undeniably a product of many factors. At the same time, it is equally undeniable that one of these factors, and indeed one with major significance, is that of public confusion (Hu 2008; Seiders and Petty 2004; Flatt 2011). Dietary messages regarding sugar content, fat content, saturated fat content, cholesterol, micronutrients, energy intake, food processing, fibre, gut health, essential fatty acids, and numerous others have created what to most is a cacophony of advice which drowns out the primary bottom line that is the caloric balance. Tempting as it may be to pin down this confused and confusing messaging on sciolist media and opportunistic commercial enterprises, it would be dishonest to fail to recognize the role of the scientific and the academic communities in it.

In the present article I would like to highlight some of the issues which are only all too common in dietetics research and its communication to the media and, ultimately, the key distal target group, that is the general public. I begin with a representative example, namely the work of Hernández-González et al. (2021) entitled “Timing of chocolate intake affects hunger, substrate oxidation, and microbiota: A randomized controlled trial” which appeared in a recent issue of The FASEB Journal^1^ and which has been widely reported on in the mainstream media. In particular, I summarize my disappointment and concern with the quality the aforementioned work and thus, by extension, with the reviewing process which has allowed a whole series of flawed conclusions and erroneous claims to be published. For the sake of brevity, on the technical side, I restrict my attention to a few most glaring issues which serve to illustrate my first point. Following on, I then turn my attention to the aforementioned manuscript handling process and discuss how its mismanagement facilitates the spread of misguided information and prevents the essential self-correcting aspect of science from taking place.

## Main body

### Technical concerns

In this section I would like to bring to attention to the kinds of methodological flaws and dubious interpretations of findings that are worryingly often found in dietetics research, using a prominently publicized work of Hernández-González et al. (2021) on the effects of chocolate intake timing on energy balance and body mass.

The authors’ discussion of results paints a rather misleading picture from the very start, the second sentence in the *Results* section stating that:

despite these **extra** [my emphasis] calories added by chocolate, they did not gain significant body weight when eating *ad libitum* within each condition. Of interest, females reduced waist circumference when having chocolate in the morning.

giving the impression that the participants actually increased their caloric intake. Yet, shortly thereafter we learn that this was not the case and that morning chocolate consumption:

as compared to the non-chocolate condition …spontaneously reduced their *ad libitum* energy intake by 16%.

In other words, the finding is that a small increase in net energy intake over a short period of time did not effect a significant weight gain – a finding which is hardly surprising, and certainly not as intriguing as suggested by the authors’ claim that “despite…extra calories…they did not gain significant body weight”. Many of the authors’ other findings, once stripped of obfuscating language and technical jargon, reduce to similarly trivial observations.

The authors then turn their attention to the observed caloric compensation and speculate firstly that the compensation may be due to “specific components of chocolate, such as epicatechin”, then adding the possibility of a contributory effect of the “macronutrient composition of milk chocolate”. Yet, their trial offers no means of providing evidence in support of either hypothesis, owing to the lack of appropriate control conditions. To support the former hypothesis, there should have been a period during which instead of chocolate, an energy, macronutrient, timing, appeal, and expected “healthiness” perception matched meal was consumed; to support the latter, an energy, timing, appeal, and expected “healthiness” perception, but not macronutrient, matched meal. The control condition, of no chocolate intake, is woefully inadequate, with the authors failing to control for numerous confounding factors. Moreover, probably the most straightforward explanation of the observed effect, is not even considered: namely, that the compensation may be due to a conscious effort caused by the participants’ awareness of the unusual dietary practice that they were asked to observe at the onset of the day and the unfavourable “healthiness’ associations with chocolate (“fattening”, high calorie, high fat, high sugar, processed, etc.). The trial was by design *de facto* unblinded. Had the authors attempted to analyse the results using an approach designed for such trials (Arandjelović 2012), they would have realized the inherent flaws of their design, if these were not apparent already. Thus, the authors’ conclusion that:

The intake of a rather high amount of chocolate (100 g) concentrated in a narrow (1 h) timing window in the morning could help to burn body fat and to decrease glucose levels in postmenopausal women.

is at best grossly misleading and rather irresponsible. It can hardly be surprising that, reporting on the study, The Boston Herald lead with “Eating milk chocolate may help burn fat”, The Harvard Gazette with “Eat the chocolate, lose the weight?”, The Sun with “Eating CHOCOLATE for breakfast can supercharge your weight loss, scientists discover”, The India Times with “Eating Milk Chocolate After Waking Up Could Help With Weight Loss”, The Mirror with “Eating chocolate for breakfast can help you lose weight, scientists say”, etc.

What makes the aforementioned daring conclusion drawn by the authors even more unacceptable, is that it is based on mere *two weeks* of observation. There is little to surprise in the fact that the authors’ claims were readily picked up by what looks like an endless stream of media outlets^2^ . I cannot help but be reminded of Zeno, who rejected the possibility of motion on the basis of thought experiments (Anglin 1994). How does one, upon reaching an apparently absurd conclusion, not stand back for a moment and re-examine the process that led to it? How do the gatekeepers of scientific publications not do the same?

I also must comment on the following passage from the article:

Considering the so-called 3,500-calorie rule that estimates that increasing caloric intake by 500 kcal per day, or 3500 kcal per week would result in 1 lb of weight gain per week, these postmenopausal females should have gained more than 2 lb ( ∼ 1 kg) in each chocolate intervention (2 × 7 × 542 kcal = 7588 kcal) if there would be no differences in ad libitum intake or energy expenditure.

Putting aside that the reference cited in support, to the best of my reading, makes no mention of this “rule”, the “rule” claimed is no such thing at all but rather a reflection of a serious misapprehension. The quantity, 3500 kcal, comes from considering the energy content of a pound of *adipose tissue*. The arithmetic is thus: since approximately 85% of adipose tissue is fat, the pound being equal to about 454 g and the rough caloric content of a gram of fat being 9 kcal, the approximate energy content of a pound of adipose tissue is 0.85×454×9 kcal ≈3473 kcal, i.e. about 3500 kcal. Note that this simple calculation refers to adipose tissue only – the energy content of other tissue types (e.g. contractile muscle tissue or glycogen) is significantly different. Given that the gain of adipose tissue only would be an extraordinary occurrence even in the most extreme pathological cases, the authors’ claims as regards the expected body weight increase are erroneous on this account alone already. However, there is more: the energy content of tissue is not the same as the total energy required to *synthesise* this tissue, which is a distinction the authors fail to make. And then there are faecal and urinary losses (Norgan and Durnin 1980), etc.

To conclude, there is no doubt that the primary responsibility for the content of an article lies with its authors and it is at them that the bulk of my criticism is aimed. However, it is equally true that a certain share of responsibility lies with the journal that publishes the article, its editors and reviewers. Arguably, this is particularly the case when it is clear that the authors’ claims are likely taken as authoritative advice regarding a health issue (Arandjelović 2022). As many have emphasised a number of times in the past (Arandjelović 2021; Cooper et al. 2021), the general public continues to show a high degree of faith in scientists, and this faith demands responsible behaviour – if it is betrayed the situation may become perilous indeed (Arandjelović 2016). Hence, each of us has the duty to ensure rigour both in terms of technical methodology and communication of ideas and findings – which leads us to the issue at the crux of the next section.

### Individual and systemic responsibility

None of the issues highlighted in the previous section are particularly subtle. Hence, a young and inexperienced researcher, or indeed a lay but otherwise educated member of the public, could be forgiven for wondering how it is possible that such gross errors could have escaped two supposedly expert reviewers as well as multiple editors (usually an Associate Editor, an Area Editor in Chief, and the Editor in Chief); and let us remember what only all too often appears to get forgotten, that it is the editors with whom the ultimate decision on the publication of an article rests, with the reviewers’ role being purely advisory (Arandjelović 2017).

Yet, in principle, the error checking and correcting process does not end with the publication of an article – the system, as envisaged, is more robust than that. In particular, I am referring to the importance of follow-up commentary and, importantly, criticisms of published research in the form of letters to the editor – which Spodick (1996) describes as “essential for peer review” in “evidence-based medicine”. Dkhar (2018) offers a good summary of the key reasons for this all but universal appreciation of letters to the editor:

International Committee of Medical Journal Editors (ICMJE) recommends publication of these letters in journals together with their answers. The International Committee of Medical Journal Editors has declared that all biomedical journals should have such a section because the absence of one ‘denies readers the possibility of responding to articles in the same journal that published the original work.’

I would superadd two more quotes from ICMJE’s recommendations^3^, namely:

Matters of debate are best handled as letters to the editor,…

and, regarding expressions of concern:

…they should be prominently labelled, appear on an electronic or numbered print page that is included in an electronic or a print Table of Contents to ensure proper indexing, and include in their heading the title of the original article.

Similar points, again stressing the importance of post-publication review and discussion are made by Tierney et al. (2015):

Letters to the editor serve two main purposes; post-publication peer review and sharing experiences with fellow readers. Both are equally important in maintaining journals’ high standards. Indexing needs to be improved otherwise valuable comment does not endure while the original manuscript’s message lives on.

Hence, it should be a matter of serious concern for the entire community what transpired when I tried to express my concerns, summarized in the previous section, to The FASEB Journal. Following my unsuccessful attempts at finding out how to submit a letter to the editor, I contacted the journal’s administrative office who informed me that I can send it to them, and that they would then forward it to the Editor in Chief. After doing so a few days later, I promptly received a response from the Managing Editor who informed me that the journal does not publish letters to the editor after all. Clearly, this policy (in so much that it *is* a policy, considering the original response I received and seeing that to the best of knowledge no such policy is stated anywhere on the journal’s web site) stands in stark opposition to ICMJE’s guidance, leaving The FASEB Journal, and thus also any problematic, questionable, or misleading content published in it, out of reach of *direct* scrutiny and criticism which would be archived and permanently associated with the original article. In a single move, The FASEB Journal has removed, to use the very title of the paper by Winker and Fontanarosa (1999), “a forum for scientific discourse”.

### Summary

Dietetics research is only all too often riddled with methodological inadequacies, erroneous claims, and misleading interpretations of findings, uncorrected by a system which fails to uphold some of the basic principles of scientific inquiry. Standing idly by while a preventable wrongdoing takes place does not make one an innocent bystander, but a complicit party. Hence, it is the duty of individual scientists to speak out and challenge poor science, unsatisfactory publishing processes, and bombastic and misleading communication of research.

## Notes

1. The flagship publication of the Federation of American Societies for Experimental Biology (FASEB): https://faseb.onlinelibrary.wiley.com/journal/15306860.

2. I am forced to weaken my judgement from what it would have otherwise read, by the authors’ use of the word “could”, which pushes their conclusion into the realms that scientists should not venture into, namely that of Popper’s unfalsifiability.

3. See http://www.icmje.org/icmje-recommendations.pdf
